# Reproducibility of the 75 g oral glucose tolerance test for the diagnosis of gestational diabetes mellitus in a sub-Saharan African population

**DOI:** 10.1186/s13104-017-2944-7

**Published:** 2017-11-28

**Authors:** Yvonne Nangeh Munang, Jean Jacques Noubiap, Celestin Danwang, Julius Dohbit Sama, Marcel Azabji-Kenfack, Jean Claude Mbanya, Eugene Sobngwi

**Affiliations:** 1 Department of Internal Medicine and Specialties, Faculty of Medicine and Biomedical Sciences, University of Yaoundé I, Yaoundé, Cameroon; 2 Department of Medicine, Groote Schuur Hospital and University of Cape Town, Cape Town, South Africa; 3 Department of Surgery and Specialties, Faculty of Medicine and Biomedical Sciences, University of Yaoundé I, Yaoundé, Cameroon; 4 Department of Obstetrics and Gynecology, Faculty of Medicine and Biomedical Sciences, University of Yaoundé I, Yaoundé, Cameroon; 5 Department of Physiology, Faculty of Medicine and Biomedical Sciences, University of Yaoundé I, Yaoundé, Cameroon; 6 Laboratory for Molecular Medicine and Metabolism, Biotechnology Center, University of Yaoundé I, Yaoundé, Cameroon; 7 National Obesity Center, Yaoundé Central Hospital, Yaoundé, Cameroon; 8 National Obesity Center, Yaoundé Central Hospital and Faculty of Medicine and Biomedical Sciences, University of Yaoundé 1, Yaoundé, Cameroon

**Keywords:** Gestational diabetes, Oral glucose tolerance test, Reproducibility, Cameroon, Africa

## Abstract

**Objective:**

To evaluate the reproducibility of the 75 g oral glucose tolerance test and factors associated with non-reproducible results in Cameroonian pregnant women.

**Results:**

Twenty-seven of the 84 participants (32.1%) who did the first oral glucose tolerance test were diagnosed with gestational diabetes mellitus. There was no difference between the means of the glycaemic responses at T0 (*p* = 0.64), T30 (*p* = 0.08), T60 (*p* = 0.86), T90 (*p* = 0.51), and T120 (*p* = 0.34) between the two oral glucose tolerance test. Age (*p* = 0.001) and BMI (*p* = 0.001) were significantly associated with non-reproducible results. The reproducibility of the oral glucose tolerance test in this study was 74.2%, and the kappa statistic’s 0.46. In conclusion, the results of the oral glucose tolerance test were reproducible in only 74.2% of pregnant women in this study. This highlights that a single oral glucose tolerance test for the diagnosis of gestational diabetes mellitus should be interpreted with caution.

**Electronic supplementary material:**

The online version of this article (10.1186/s13104-017-2944-7) contains supplementary material, which is available to authorized users.

## Introduction

Gestational diabetes mellitus (GDM) is defined as any degree of glucose intolerance occurring or recognised for the first time during pregnancy [1]. GDM is a major global public health problem owing to its high prevalence and adverse outcomes in the mother and the foetus or neonate. The International Diabetes Federation (IDF) estimated that 21.4 million live births were affected with hyperglycaemia in pregnancy in 2013 globally, with GDM accounting for 90% of cases [2]. The prevalence of GDM varies widely depending on the population and diagnostic criteria used. Ninety percent of all cases of hyperglycemia in pregnancy occur in low- and middle-income countries, with sub-Saharan Africa ranking second after South-East Asia [2]. A recent meta-analysis has estimated the prevalence of GDM in sub-Saharan Africa at 5.1% of all pregnancies, and 14% in high-risk pregnant women [3]. And depending on diagnostic criteria, it ranged from 5 to 17% in Cameroon in 2010 (unpublished data from E. Sobngwi).

GDM is associated with adverse maternal complications including hypertension, preeclampsia, infections, post-partum haemorrhage, obstructed labour and increased operative intervention, future GDM and diabetes mellitus in the long term [1, 4–8]. In the foetus and neonates it is associated with miscarriage, stillbirth, preterm births, macrosomia, hydramnios, congenital anomalies, metabolic abnormalities, respiratory distress syndrome, birth injuries and subsequent childhood and adolescent obesity [1, 8].

Appropriate diagnosis and management of GDM are crucial to reduce the risk of perinatal and long-term complications [9]. Several diagnosing criteria for GDM are used worldwide, including those from the World Health Organization (WHO) [10], the America Diabetes Association (ADA) [11] and the International Association of Diabetes and Pregnancy Study Groups (IADPSG) [12]. While the recommended diagnostic test is the oral glucose tolerance test (OGTT), diagnostic criteria differ in the target population for screening (universal or only high-risk women), gestational age at screening, loading dose for the OGTT and cut-off levels of plasma glucose.

One of the major limitations of the OGTT is its low reproducibility as demonstrated in non-African pregnant and non-pregnant women [13]. The current study aimed to evaluate the reproducibility of the 75 g OGTT in a Cameroonian population. We were particularly interested in describing the variations of the 75 g OGTT and determining factors associated with non-reproducible results.

## Main text

### Methods

#### Setting and study population

This prospective study was conducted from December 2012 to May 2013 in two antenatal clinics of the Integrated Health Centres of Nkwen and Azire in the South West region of Cameroon. The study population consisted of pregnant women between 24 and 28 weeks of gestation, aged more than 18 year old, attending antenatal care at these centres and who accepted to participate in the study. Women with a known history of diabetes mellitus, as well as those with long term medical treatment, on steroids and beta-mimetics or presenting with any medical complications (like severe malaria, hyperemesis gravidarum, acute hepatitis, pyelonephritis) were excluded from the study.

#### Data collection

For each subject, data were collected in two steps:

Step 1 consisted of an initial screening of all participants using fasting blood glucose (FBG). FBG was done on capillary whole blood after a fasting period of at least 8 h using the Accu-Chek^®^ Compact Plus glucometer (F. Hoffmann-La Roche AG, Basel, Switzerland). In order to achieve reliable diagnosis and classification of hyperglycaemia in pregnancy, it is recommended to measure venous plasma or serum glucose using an enzymatic method with high accuracy and precision [14, 15]. Unfortunately these conditions for accuracy are sophisticated and cannot be met in most of peripheral hospitals in low- and middle-countries like Cameroon. Since equivalence formulae between venous plasma and capillary glucose levels exist, we used capillary whole blood values [14, 15]. Those who had an FBG at or above 0.92 mg/dL had a positive screening test. The study population was then divided in two groups: Group A consisted of all women who were positive on initial screening, while Group B consisted of a number of women randomly selected from those who were negative on screening. Selected women in Groups A and B were invited for a 75 g OGTT within a week after initial screening.

In Step 2, a 75 g OGTT was performed in women in Groups A and B. The OGTT was done twice for each participant. Participants were asked to respect the following instructions before coming for the test: (i) have an unrestricted diet in carbohydrates in the days preceding the test; (ii) carry on their physical activities as usual; (iii) stay at home if they were suffering from any illness; (iv) have an overnight fast of 8–14 h [16]. No change in participants’ behaviors with respect to eating and physical activity was noted throughout the study. A first 75 g OGTT was carried out according to standard protocol [16], and 7 days later, a second 75 g OGTT was done. Both OGTT were done in the same standard conditions, using the same glucometer used for all blood glucose measurements done throughout this study. We measured FBG and repeated measurements successively 30 min (T30), 60 min (T60), 90 min (T90) and 120 min (T120) after the 75 g glucose load. We used the most recent diagnostic criteria for the 75 g OGTT published by the International Association of Diabetes and Pregnancy Study Group. The test was considered as positive if any of the values met the following criteria: FBG ≥ 92 mg/dL; 1-h glycaemic response ≥ 180 mg/dL; 2-h glycaemic response ≥ 153 mg/dL [12].

The same day of the second test, additional data were collected including relevant past medical, obstetric and gynaecological history, nutritional and physical exercise habits, anthropomorphic measurements and blood pressure. For all participants, we measured weight in light clothes with a Seca Scale balance to the nearest 0.1 kg, height with a calibrated stadiometer to the nearest 0.5 cm. Body mass index (BMI) was calculated as weight (in kg) divided by the square of height (in m^2^). We measured resting blood pressure using standardized procedures with the participant in a seated position, and after at least 10 min rest with a validated automated blood pressure measuring device, the Omron HEM-757 (Omron Corporation, Tokyo, Japan). The mean of two measures performed at least 3 min apart was used for all analyses.

#### Statistical analyses

Data were coded, entered and analyzed using the Statistical Package for Social Science (SPSS) version 20.0 for Windows (IBM Corp. Released 2011. IBM SPSS Statistics for Windows, Version 20.0. Armonk, NY: IBM Corp.). Data were expressed as counts and frequencies, means with standard deviations. Frequencies were compared using the Chi squared test, means were compared using the samples T test, inter observer variability was assessed using the Cohen Kappa Statistics and partial correlations. A *p* value < 0.05 was considered statistically significant.

We evaluated the variation between the two oral glucose tolerance tests, and the correlation between the values of glycaemic responses in the two tests. Furthermore, the Bland–Altman analysis was used to calculate the coefficient of repeatability and the bias for glycaemic responses at T0, T30, T60, T90 and T120.

To determine potential factors associated with variability of glycaemic response we classified participants into four groups as follows, Group 1: normal results at each testing (Negative–Negative); Group 2: initially normal, then abnormal at retesting (Negative–Positive); Group 3: initially abnormal results, then normal at retesting (Positive–Negative) and Group 4: abnormal results on both testing (Positive–Positive). Characteristics of participants with discordant results were compared with those with concordant results in order to identify factors associated with variability in results.

We calculated the reproducibility of the 75 g OGTT by summing up the number of cases that presented with identical results on first testing and retesting and divided by the overall number of participants who completed the OGTT twice. We tested the level of agreement of the results of the OGTT on test and retest, using the Cohen Kappa’s statistics calculated as follows:

$$\upkappa = \frac{{\kappa_{0} - \kappa_{e} }}{{1 - \kappa_{e } }}$$

$$\kappa_{0} = {\text{ the}}\;{\text{observed}}\;{\text{agreement }} = \frac{a + d}{a + b + c + d}$$

$$\begin{aligned}{\text{and}}\;\kappa_{e} & = {\text{the}}\;{\text{expected}}\;{\text{agreement }} \\ & = \left( {\frac{c + d}{a + b + c + d}} \right)\left( {\frac{b + d}{a + b + c + d}} \right) \\ & \quad + \left( {\frac{a + b}{a + b + c + d}} \right)\left( {\frac{a + c}{a + b + c + d}} \right) \end{aligned}$$

a = number of normal results at each testing (Negative–Negative); b = number of results initially normal, then abnormal at retesting (Negative–Positive); c = number of results initially abnormal results, then normal at retesting (Positive–Negative); and d = number of abnormal results on both testing (Positive–Positive).

The sample size was estimated using the following formula:

$${\text{N }} = \left( { 2/{\text{d2}}} \right) *{\text{Cp}}$$

d = standardised difference = target difference/standard deviation

Considering statistical significance at 5% and the power at 80% with Cp being a constant. We estimated the number of women to be screened at 240 but in other to increase statistical power, we screened 400 women.

### Results

A total of 978 women attended antenatal care (ANC) consultations in the Integrated Health Centers (IHC) of Nkwen and Azire during our study period. Of these women, 426 were between 24 and 28 weeks of gestation and eligible, and 400 accepted to participate in the study, giving a participation of rate of 93.9%. Thirty (75%) participants were positive on screening and 25 of them accepted to do the OGTT twice. Of all the participants who were negative on screening, 60 were randomly selected to do the OGTT twice. We had a total of 85 participants who accepted to do the OGTT twice but only 70 of them completed it giving a completion rate of 82%.

The mean age of the participants was 26 ± 5 years. The most represented age group was 25–34 and the mean gestational age was 25 weeks ± 5 days. In this study, 5.7% of participants were multiparous while 30% were nulliparous (Table 1).

**Table 1:** General characteristics of the study population

Characteristics	Frequency	Percentage (%)
Age		
15–24	148	37
25–34	228	57
35–45	24	6
Level of education		
Primary	137	24.2
Secondary	195	48.8
University	68	17
Parity		
0	120	30
1–3	257	64.3
≥ 4	23	5.7
Gestational age (weeks)		
24	76	19
25	92	23
26	64	16
27	132	33
28	40	10
Risk factors for GDM		
Past history of macrosomia	11	2.8
Past history of GDM	14	2.0
Obesity before pregnancy	3	0.8

*GDM* gestational diabetes mellitus

#### Prevalence of gestational diabetes mellitus

Prevalence for GDM was calculated using the results of the first OGTT. A total of 84 participants did the first OGTT. Twenty-seven of these women were diagnosed with GDM, giving a prevalence of 32.1%.

#### Variations of the 75 g oral glucose tolerance test

**Fig. 1:**
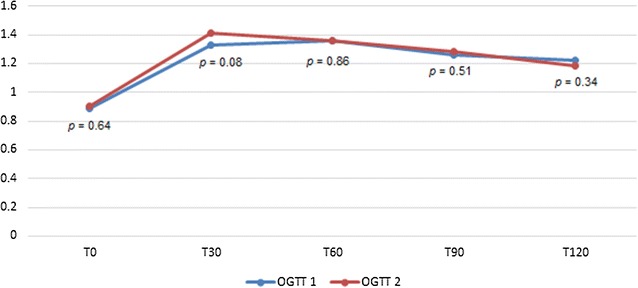
Comparison of the means of glycaemic responses for OGTT 1 and OGTT2

We evaluated the variation between the two OGTT, and the correlation between the values of glycaemic responses in the two tests. We found no difference between the means of the glycaemic responses at T0 (*p* = 0.64), T30 (*p* = 0.08), T60 (*p* = 0.86), T90 (*p* = 0.51), and T120 (*p* = 0.34) between the two OGTT (Fig. 1). We found a positive correlation between the values of glycaemic responses in the two OGTT at T0 (r = 0.55, *p* = 0.023), T60 (r = 0.42, *p* = 0.001), and T120 (r = 0.355, *p* = 0.003) (Additional file 1: Figure S1).

**Fig. 2:**
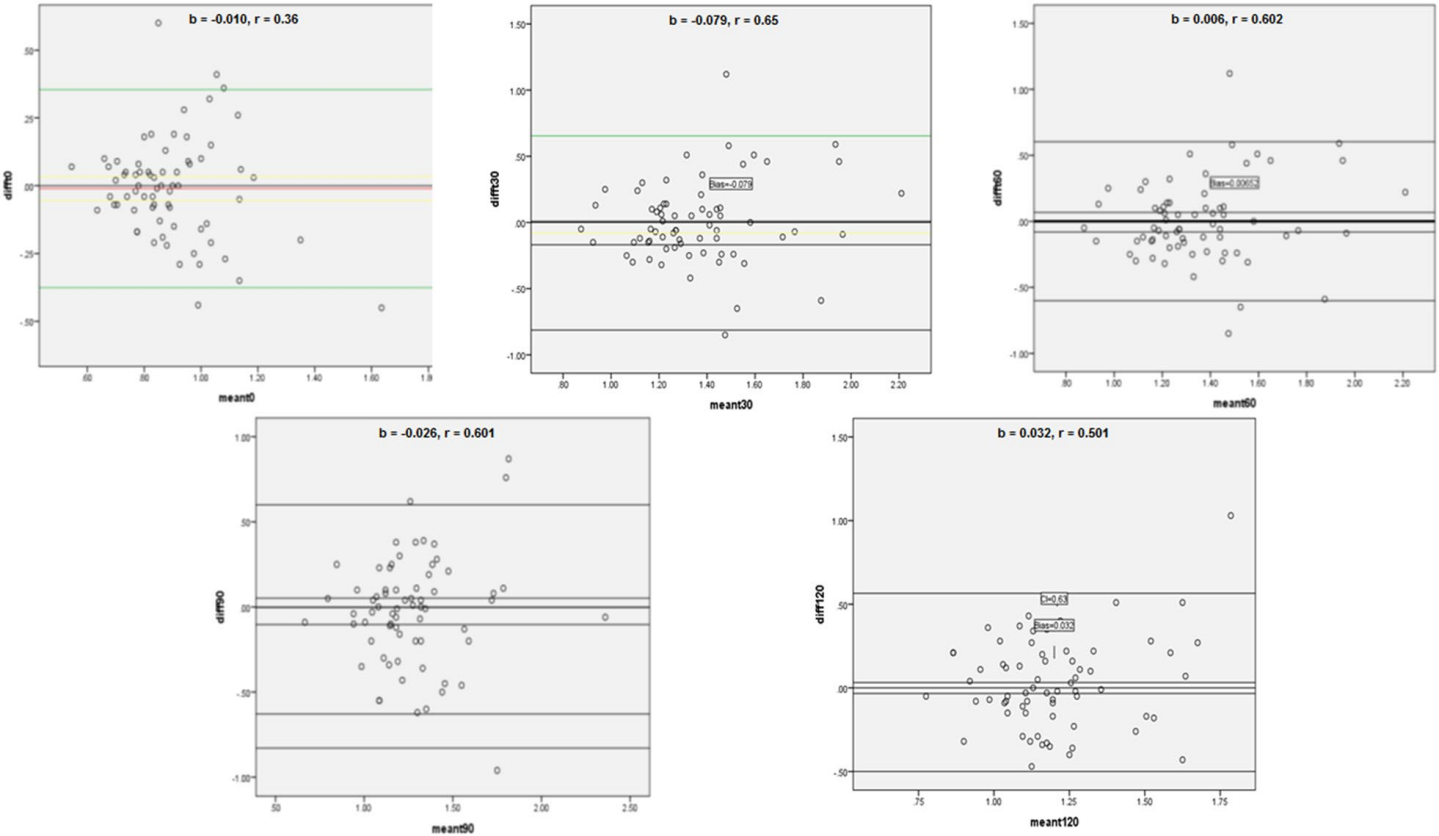
Bland Altman analysis of glycaemic response at T0, T30, T60, T90 and T120 for OGTT 1 and OGTT 2

Using Bland–Altman analysis, the bias and the coefficients of repeatability were respectively − 0.010 and 0.36 at T0, − 0.079 and 0.65 at T30, 0.006 and 0.602 at T60, − 0.026 and 0.601 at T90 and 0.032 and 0.501 at T120 (Fig. 2).

#### Reproducibility of OGTT and factors associated with non-reproducible results

We found that 48.6% of participants were negative on both testing, 11.4% were initially negative, then positive on retesting, 14.3% were initially positive, then negative on retesting and 25.7% were positive on both testing (Additional file 2: Table S1). The reproducibility of the OGTT in this study was 74.2%, and the kappa statistic’s 0.46. By comparing characteristics of participants with discordant results with those with concordant results, we found that age (*p* = 0.001) and BMI (*p* = 0.001) were significantly associated with non-reproducible results. There was no association between gestational age (*p* = 0.143), parity (*p* = 0.854), level of education (*p* = 0.535), frequency of fruit consumption (*p* = 0.173), frequency of vegetable consumption (*p* = 0.745), minutes spent walking per day (*p* = 0.819), number of sporting activities per week (*p* = 0.647), blood pressure (*p* = 0.118) and non-reproducible results.

### Conclusion

The 75 g OGTT results were reproducible in only 74.2% of pregnant women in this study. The main factors associated with non-reproducible results were maternal age and BMI. These findings highlight that a single oral glucose tolerance test for the diagnosis of gestational diabetes mellitus should be interpreted with caution.

### Limitations

Our study is mainly limited by its relative sample size which lessens the generalizability of our findings. Another limitation of this study is the measurement of blood glucose on capillary blood samples using glucometer. For better accuracy, it is recommended to measure venous plasma or serum glucose using enzymatic methods. However, this study was conducted in a resource-limited setting with no access to standard diagnostic procedures. Although there are equivalence formulae between venous plasma and capillary glucose levels, venous plasma measurement remains the most reliable method. Thus, the result of our study cannot be compared directly with those done using serum glucose.

## Additional files

**Additional file 1: Figure S1.** Correlations between glycaemic responses at T0, T30, T60, T90 and T120 for OGTT 1 and OGTT 2.

**Additional file 2: Table S1.** Classification of results of the two oral glucose tolerance tests.

## Abbreviations

OGTT: oral glucose tolerance test
GDM: gestational diabetes mellitus
IDF: International Diabetes Federation
WHO: World Health Organization
ADA: American Diabetes Association
IADPSG: International Association of Diabetes and Pregnancy Study Groups
