# Supplementary figures and images for: Reproducibility of the 75 g oral glucose tolerance test for the diagnosis of gestational diabetes mellitus in a sub-Saharan African population
Source: BMC Res Notes. 2017 Nov 28;10:622. doi: 10.1186/s13104-017-2944-7 (PMC5704589; doi:10.1186/s13104-017-2944-7)

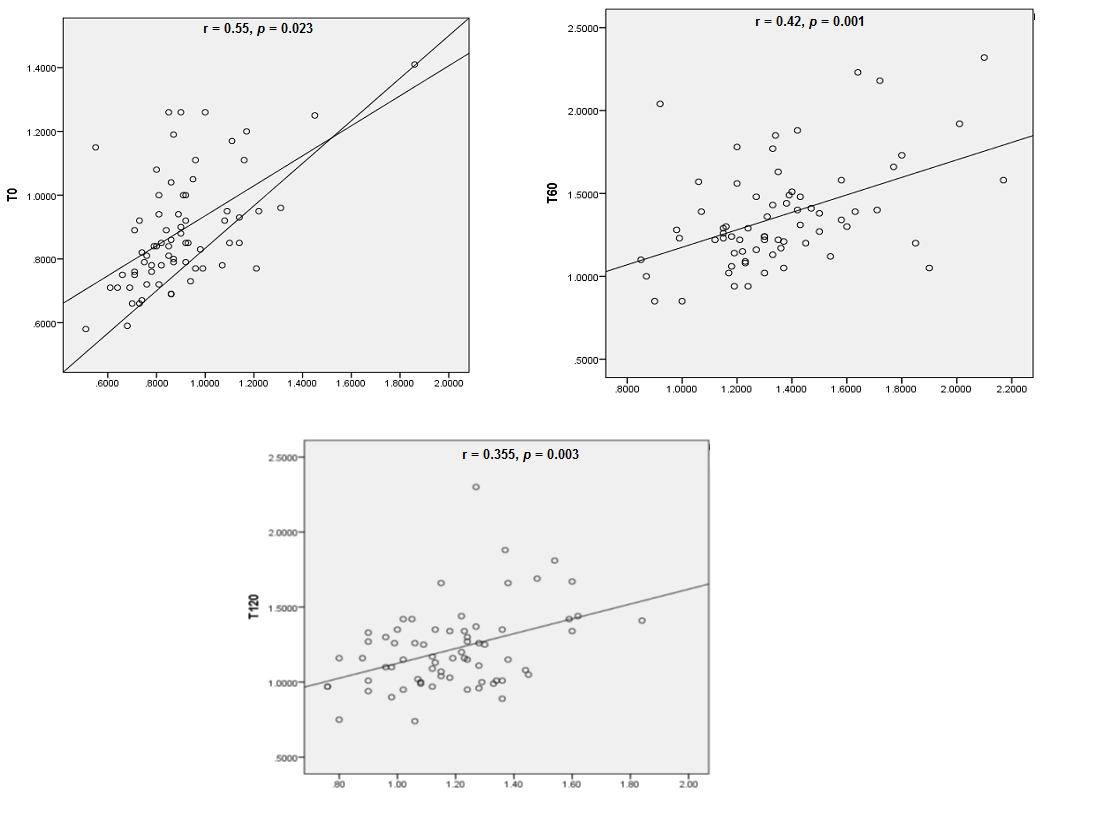

Supplement: Supplementary file 1 — Additional file 1: Figure S1. Correlations between glycaemic responses at T0, T30, T60, T90 and T120 for OGTT 1 and OGTT 2. [file 13104_2017_2944_MOESM1_ESM.tif]
