# Supplementary material for: Reproducibility of the 75 g oral glucose tolerance test for the diagnosis of gestational diabetes mellitus in a sub-Saharan African population
Source: BMC Res Notes. 2017 Nov 28;10:622. doi: 10.1186/s13104-017-2944-7 (PMC5704589; doi:10.1186/s13104-017-2944-7)
Supplement: Supplementary file 2 — Additional file 2: Table S1. Classification of results of the two oral glucose tolerance tests. [file 13104_2017_2944_MOESM2_ESM.docx]

**Table S1. Classification of results of the two oral glucose tolerance tests**

| **Classification** | **Frequency** | **Percentage** |
| --- | --- | --- |
| Negative-Negative | 34 | 48.6 |
| Negative-Positive | 8 | 11.4 |
| Positive-Negative | 10 | 14.3 |
| Positive-Positive | 18 | 25.7 |
| **Total** | **70** | **100** |
